# Functional Mobility Training With a Powered Knee and Ankle Prosthesis

**DOI:** 10.3389/fresc.2022.790538

**Published:** 2022-04-11

**Authors:** Suzanne B. Finucane, Levi J. Hargrove, Ann M. Simon

**Affiliations:** ^1^Center for Bionic Medicine, Shirley Ryan Abilitylab, Chicago, IL, United States; ^2^Department of Physical Medicine and Rehabilitation, Northwestern University, Chicago, IL, United States; ^3^Department of Biomedical Engineering, Northwestern University, Chicago, IL, United States

**Keywords:** physical therapy, above-knee amputation, ambulation, robotic prosthesis, rehabilitation, artificial leg, prosthesis training, transfemoral amputation

## Abstract

Limb loss at the transfemoral or knee disarticulation level results in a significant decrease in mobility. Powered lower limb prostheses have the potential to provide increased functional mobility and return individuals to activities of daily living that are limited due to their amputation. Providing power at the knee and/or ankle, new and innovative training is required for the amputee and the clinician to understand the capabilities of these advanced devices. This protocol for functional mobility training with a powered knee and ankle prosthesis was developed while training 30 participants with a unilateral transfemoral or knee disarticulation amputation at a nationally ranked physical medicine and rehabilitation research hospital. Participants received instruction for level-ground walking, stair climbing, incline walking, and sit-to-stand transitions. A therapist provided specific training for each mode including verbal, visual, and tactile cueing along with patient education on the functionality of the device. The primary outcome measure was the ability of each participant to demonstrate independence with walking and sit-to-stand transitions along with modified independence for stair climbing and incline walking due to the use of a handrail. Every individual was successful in comfortable ambulation of level-ground walking and 27 out of 30 were successful in all other functional modes after participating in 1–3 sessions of 1–2 h in length (3 terminated their participation before attempting all activities). As these prosthetic devices continue to advance, therapy techniques must advance as well, and this paper serves as education on new training techniques that can provide amputees with the best possible tools to take advantage of these powered devices to achieve their desired clinical outcomes.

## Introduction

Individuals with lower-limb loss at the transfemoral or knee disarticulation level lose a significant amount of mobility due to missing both an ankle and knee joint (1–3). Various daily activities are affected including walking in the community, negotiating obstacles within their home or work environment, navigating curbs or ramps, and transitioning to and from a seated position. These routine tasks are very challenging for most individuals because commercially available prosthetic joints are mechanically passive devices and cannot provide joint power similar to anatomical joints. During walking, passive ankle joints cannot provide ankle push-off power in a late stance and passive knees cannot actively extend during the swing phase, thus, requiring individuals to provide active hip flexion to advance the prosthetic limb. While ascending steps, ascending an incline, or standing up, unilateral transfemoral amputees must rely heavily on their non-amputated limb and may resort to other compensatory movements such as increased upper extremity support (4, 5) or excessive vaulting (6, 7). As a result, these individuals expend more energy and have slower walking speeds than persons without an amputation (8, 9).

Prosthetic knees and ankles that have the capability of restoring power at the missing joints are becoming commercially available (10, 11) and with several more in development (12–20). The availability of power can allow for more normative gait kinematics (15, 21, 22) and can re-introduce an individual to activities they may not have completed since prior to their amputation including climbing stairs in a reciprocal manner (23–26), ambulating up and down, long and/or steep inclines with confidence (25, 27, 28), and transitioning to standing with more equal weight bearing (29, 30). The addition of power at the knee joint may reduce the occurrence of overuse injuries that occur after long-term prosthesis use. Providing the user with active powered plantarflexion during ambulation has the potential to decrease hip joint effort on the prosthetic side (31). An overarching goal for powered devices is to utilize both lower extremities more equally for daily activities. Achieving this goal may allow users to prevent further musculoskeletal injuries, as well as improve their strength, balance, and postural stability.

While the promise of powered prostheses is abundant, clinical instruction and training are needed to provide amputees with the best outcomes possible and enable them to utilize these devices to their fullest potential. Rehabilitation of transfemoral amputees varies greatly among therapists and rehabilitation facilities (32). This variation may be due to differences in patient populations across facilities, etiology, availability of therapy equipment, clinical skills and education of staff on the various devices, and accessibility to prosthetists and manufacturers for extended device training. While active devices can restore additional functional activities (e.g., reciprocal stair climbing), learning to incorporate all these features and optimize ambulation requires user and clinician education.

Educational materials are necessary to instruct patients on how to properly use these devices. For example, the manufacturer suggested training techniques for the Ossur Power Knee are divided into levels: initial training includes walking mode, intermediate training includes stair and ramp descent modes, and advanced training includes stair ascent mode (10). One Power Knee study cited 16 h of training to allow users to accomplish sit to stand transitions, stair ascent/descent, incline walking, and walking over uneven terrain (24), while another study showed that transfemoral amputees first fit to the Ossur Power Knee achieved functional mobility milestones in less time than those who were fitted first to a non-powered knee devices (16, 33). Studies investigating transtibial amputees' functional mobility during incline walking and stair climbing with the emPOWER (formerly the BiOM)-powered ankle indicated that more focused and device-specific gait training is recommended (31, 34, 35). Introducing clinicians to these devices with more opportunities for appropriate education and training will likely have a positive impact on physical therapy practice, goal setting, compliance of wear, and use of advanced devices for patients with transfemoral amputations (36).

The purpose of this paper is to help fill the gap in education regarding instructing transfemoral amputees on the use and functionality of a powered knee and ankle prosthesis. These techniques and tools were developed during the training of thirty transfemoral and knee disarticulation amputee users over the last 10 years at a nationally ranked physical medicine and rehabilitation research hospital. The training was designed to meet the goals of independent ambulation through all functional mobility modes including level-ground walking, incline walking, stair ascent and descent, and sit-to-stand and stand-to-sit transitions within a rehabilitation setting.

## Methods

Thirty patients (Table 1) who have had a unilateral transfemoral or knee disarticulation amputation participated. All patients provided written informed consent as approved by the Northwestern University Institutional Review Board. Individuals were independent in ambulation for level-ground, inclines, and stairs with their current device and classified as varied cadence community ambulators (centers for Medicare & Medicaid Services K3 and K4 level). Each subject was evaluated by a certified prosthetist and either used their clinically prescribed socket or duplication of their home socket for use during training sessions. If necessary, and often due to the added weight of the device, adjustments were made to the suspension of the device either by socket modifications or the addition of socks or a suspension belt.

**Table 1:** Participant demographics and predicate device description.

**ID**	**Years post-amputation**	**Gender**	**Etiology**	**Age** **(yr)**	**Height** **(cm)**	**Weight** **(kg)**	**K-Level**	**Prescribed knee**	**Suspension type**
TF01	0.75	M	Right sarcoma	28	193	73	K3	Ottobock C-Leg®	Skin fit suction
TF02	1	M	Left traumatic	32	188	81	K3	Ottobock C-Leg®/X3®	Suction with TES
TF03	1	M	Left traumatic	48	195	94	K3	Ossur Rheo Knee® XC	Seal-in liner
TF04	2	M	Left sarcoma	68	177	79	K3	Ottobock C-Leg®	Skin fit suction
TF05	2	M	Right sarcoma	33	177	63	K3	Ottobock Genium™	Seal-in liner
TF06	3	M	Right trauma	38	177	91	K3	Ottobock C-Leg®	Skin fit suction
TF07	4	M	Right-Infection	31	175	79	K3	Ottobock 3R80	Seal-in liner
KD08	5	M	Right traumatic	36	180	77	K4	Ossur Total Knee®	Liner and lock
TF09	7	F	Left sarcoma	26	160	52	K4	Ottobock C-Leg®	Skin fit suction
TF10	8	M	Right sarcoma	41	183	103	K3	Freedom Innovations Plie®	Liner with lanyard
KD011	9	M	Right sarcoma	26	177	91	K4	Ottobock Genium™	Seal-in liner
TF12	11	M	Right traumatic	19	185	62	K3	Ottobock Genium™	Seal-in liner
TF13	11	M	Left sarcoma	32	193	104	K3	Ottobock C-Leg®	Seal-in liner
TF14	14	M	Left traumatic	27	175	78	K3	Ottobock C-Leg®	Seal-in liner
TF15	15	M	Left traumatic	63	165	99	K3	Ottobock Genium™	Seal-in liner
TF16	15	F	Right sarcoma	29	170	70	K3	Ottobock C-Leg®	Skin fit suction
TF17	17	M	Right traumatic	55	168	64	K3	Ottobock C-Leg®	Suction
TF18	17	F	Right sarcoma	38	170	66	K3	Ossur Mauch®	Skin fit suction
KD19	18	M	Left sarcoma	33	187	86	K4	Endolite Hydraulic	Skin fit suction
TF20	18	M	Right traumatic	55	187	82	K3	Ottobock Genium™	Liner and pin lock
TF21	19	M	Left traumatic	47	182	97	K4	Ossur Total Knee®	Seal-in liner
TF22	20	M	Right sarcoma	29	170	60	K3	Ottobock 3R016	Liner with pin
TF23	24	F	Right traumatic	50	165	62	K4	Ossur Rheo Knee®	Sub-ischial vacuum
TF24	29	F	Left sarcoma	36	170	73	K3	Freedom Innovations Plie®	Liner with pin lock
TF25	32	F	Right infection	58	175	69	K3	Ottobock 3R60	Liner and pin lock
TF26	35	F	Right sarcoma	52	163	68	K3	Ottobock C-Leg®	Seal-in liner
TF27	38	M	Right traumatic	69	175	86	K3	Ottobock C-Leg®	Sub-ischial vacuum
TF28	39	M	Left traumatic	56	189	111	K3	Ottobock 3R80	Liner with TES belt
TF29	46	M	Left traumatic	61	180	84	K3	Ossur Mauch®	Skin fit suction
TF30	47	M	Left traumatic	50	190	106	K4	Ottobock 3R80	Liner with pin lock

### Powered Knee and Ankle Prosthesis Description

The powered knee and ankle prosthesis (15) initially used for this study were designed by Vanderbilt University. The prosthesis provides powered knee flexion and extension through a range of motion from −5° (hyperextension) to 115° of flexion and powered ankle dorsiflexion and plantar flexion from 45° of plantarflexion to 25° dorsiflexion. Embedded prosthesis sensors measure knee and ankle joint angles, velocities, and motor currents, prosthesis load using a load cell, and prosthesis motion using a 6-degree of freedom inertial measurement unit, i.e., accelerometers and gyroscopes. The third generation powered knee and ankle prosthesis, with a custom carbon fiber footplate and standard foot shell, is ~4.75 kg in weight (15). The training concepts have also been applied and further developed in this study, while training users to walk on the Open Source Robotic Leg (37) and the lightweight robotic knee prosthesis (38). These same concepts can be applied to other powered lower limb devices. Each prosthesis is controlled using a finite state machine controller, and each ambulation mode is divided into four phases: early to mid-stance, late stance, swing flexion, and swing extension. Each phase provides a different prosthesis response to mimic near normal kinematics of level-ground walking, incline walking, and stair ascent and descent. The sensors that detect prosthesis transitions throughout these phases include the individual's load and the prosthesis joint positions and velocities. The specific details of how the user can interact with the device and move throughout the phases of each mode are described in detail in subsequent sections.

### Patient Training

Training begins by educating the user on the physical components of the device and the differences compared to their prescribed daily use prosthesis. The focus is to highlight the ability of the prosthesis to provide power in knee flexion and extension, and ankle plantarflexion and dorsiflexion across multiple ambulation modes. The majority of the K3/K4 level individuals, who fit the powered knee and ankle prosthesis in this paper, were able to independently traverse all modes available with the device within 3–6 h of instruction. The majority of the training session time is dedicated to adjusting prosthesis parameters to improve gait kinematics based on user and clinician feedback.

Users begin in the parallel bars with the prosthesis in standing mode. A gait belt should be used during initial safety training. In standing mode, they will be able to perform multi-directional weight shifting, including a single-limb stance, to gain confidence. Users will immediately notice the increased motion at their ankles compared to their passive device. The user is educated on the benefits of this available range of motion, including that it allows the prosthetic foot to remain flat and in contact with the ground during various ambulation modes (e.g., foot flat position during incline walking, allows the entire foot to be placed on a stair for ascending and descending steps, more comfortable sitting position and improved pre-positioning prior sit to stand transfers). For each mode, similar to the standard of care, the clinician will observe both swing and stance phases of the sound and prosthetic limbs in the frontal and sagittal planes, trunk position, and arm swing. Based on training information in this paper, clinical judgment is used to decipher between user causes for a particular gait deviation vs. a parameter adjustment to the device. Verbal and tactile instructions are given for improved symmetry, upright posture, and equal weight bearing to achieve desired outcomes before any prosthetic parameter changes. If the user is displaying any deviations of the trunk, such as lateral bending, decreased arm swing, or decreased trunk/pelvis rotation, the clinician should assess the socket fit and comfort.

#### Level-Ground Walking

##### Goals

**Figure 1:**
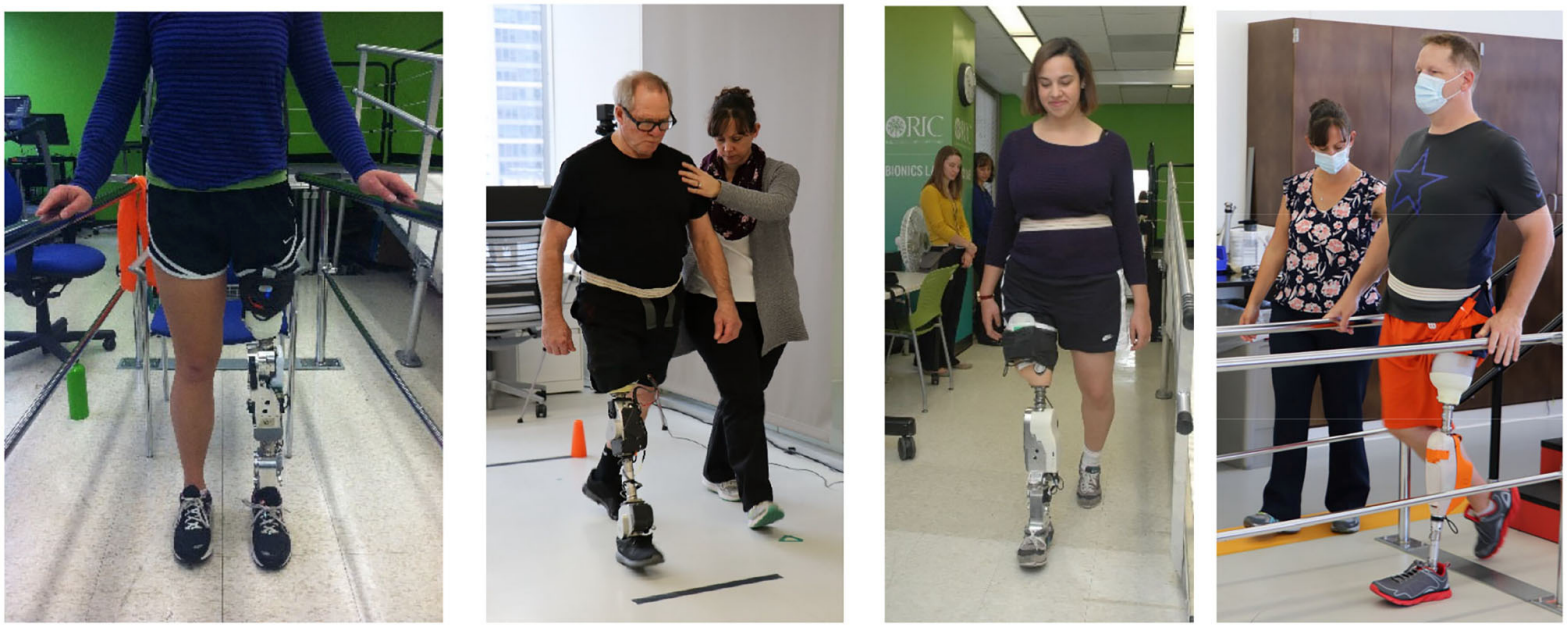
Level-ground walking with powered knee and ankle prosthesis.

Clinical goals include the ability to ambulate (Figure 1) without upper extremity support, with equal step length, arm swing, and trunk rotation, and at near desired speed without limitations or noticeable gait deviations.

##### Prosthesis Control

While walking, as the user progresses forward in stance phase over the forefoot of the prosthesis, the powered ankle dorsiflexes. When the ankle dorsiflexes past a pre-set dorsiflexion angle (usually 6–8 degrees), the prosthesis will transition to the late stance phase and begin to provide powered plantarflexion. As the user's load shifts from the prosthetic foot and onto their sound foot, the prosthesis transitions to the swing phase. The knee flexes and the ankle dorsiflexes to provide clearance and, then, actively extends to prepare the prosthesis for heel strike. Once a load is detected in the prosthesis, it will transfer into the stance phase to provide a stable knee, promote weight acceptance, and allow forward progression through stance. This cycle continues to provide steady-state level-ground walking.

##### Training

Training of level-ground walking should begin in the parallel bars to allow for upper extremity support if needed. Gait assessment is completed for both stance and swing phase of walking, while appropriate modifications are made to the powered leg parameters (Table 2) (26). During initial training, it is beneficial to instruct the user to step with their sound limb first, while providing tactile and verbal cues to increase stance time on the prosthesis, which will assist with swing initiation. While standing behind or to the side of the user, tactile cues may include physical assistance to provide force at the user's iliac crests to guide and hold their load onto the prosthesis. Additionally, verbal instruction is provided to encourage a larger sound sidestep, resulting in increased stance time on the prosthesis. The transition from late stance phase to swing phase will likely feel different from his/her prescribed device due to the ankle providing powered plantarflexion during late stance to assist with push off and swing. When necessary, a mirror can provide visual feedback to assist with prosthetic placement and to improve posture/trunk positioning.

**Table 2:** Walking mode deviations, user instruction, and prosthesis parameter adjustments.

**Mode**	**Deviation**	**User instruction**	**Prosthesis setting**
Walk	Unable to initiate swing	Cue to increase sound side step length for increased stance time on prosthesis.	Decrease pre-set minimum dorsiflexion angle for ease of swing phase initiation
	Decreased foot clearance	Cue to stand tall and utilize hip flex during swing phase.	Increase knee flexion angle during swing phase for more clearance
	Excessive hip flexion, vaulting, hip hiking, and/or circumduction	Cue and/or use a mirror to improve awareness of foot position and to decrease excessive hip motions.	N/A
	Uneven heel rise	N/A	Increase or decrease knee flexion angle during swing phase to modify heel rise
	Insufficient swing speed	N/A	Increase swing extension knee stiffness to improve swing speed
	Rapid plantar flexion at heel strike	N/A	Increase early to mid- stance ankle stiffness and/or damping

If the prosthetic knee is extending too quickly during the swing phase and resulting in a forceful terminal impact, it is important to assess the user's interaction with the prosthesis. Many users are accustomed to providing a forceful hip flexion motion to advance their prostheses. This excessive motion is no longer needed since the device can provide powered swing extension. Verbal prompts to lessen hip flexion motion, by providing awareness to the user that the prosthesis is providing adequate swing clearance and the increased hip flexion movement is not necessary, may diminish the deviation. If the excessive terminal impact continues, the knee extension parameters are adjusted to reduce the speed of swing extension. Alternatively, if the leg is not extending quickly enough, he/she may be walking quicker than the device's initial settings allow. Swing extension parameters should be adjusted to decrease swing time and accommodate the user's speed.

Decreased clearance of excessive heel rise during the swing is corrected by adjusting the swing flexion parameters. Increased hip flexion during swing or vaulting of the sound limb could also be demonstrated by users. This may be due to habit or caused by being unaccustomed to the feel of the device and its active ankle/foot mechanism. Clinical reassurance that the user has appropriate clearance during swing due to the active dorsiflexion, and/or providing visual feedback with the use of a mirror may minimize this deviation.

Demonstration of forceful heel strike and strong hip extension is commonly observed with the prescribed passive device to ensure full knee extension and foot placement for initial contact. Similar to microprocessor or other stance control passive knees aligned to allow knee flexion, further education is provided that the knee does not need to be fully extended to accept their weight at heel strike; the leg will support them when a load is detected. Once this is addressed, ankle stiffness and/or knee extension parameters in early to mid-stance can be adjusted for comfort and allow a smooth weight acceptance.

The amount and timing of powered plantarflexion should be monitored to ensure it is comfortable for the user and does not interfere with foot clearance during swing. If necessary, the amount of powered plantarflexion can be reduced during initial training. Additionally, the user may show difficulty initiating the swing phase. This can be pronounced if the user displays a shortened step length with their intact limb and decreased stance time on the prosthesis, as often seen with passive devices. Verbal and tactile cues through palpation and contact guard assist with the gait belt to guide the user to take longer sound sidesteps and increase weight bearing through stance to allow the prosthesis to swing with more natural timing. The pre-set dorsiflexion ankle angle, nominally set to 6–8 degrees of dorsiflexion, can be reduced to ease swing initiation when feedback to the user is not effective. This necessary dorsiflexion angle is what allows the prosthesis to transition to the late stance phase and for the leg to begin to provide powered plantarflexion. While training an amputee, who may be hesitant or tends to have a step-to gait with their prescribed prosthesis, decreasing the dorsiflexion angle parameter may allow ease of transitioning into swing during training. Once the user becomes more comfortable with the device and begins to show increased step length, this parameter is often adjusted back to the starting range. Once the user is walking comfortably within the parallel bars, the walking distance can be increased, and the user should be able to ambulate with decreased upper extremity support. Individuals often adapt quickly and achieve improved swing initiation in a longer walkway as they gain confidence in stance and demonstrate their ability to increase their stance time on the device. This feature of stance stability can promote increased step length and stance time, while also improving trunk/pelvic rotation and arm swing. Usually within 5–10 min of level-ground walking training, the K3/K4 level users can walk comfortably without assistance or significant gait deviations.

#### Stair Climbing

##### Goals

**Figure 2:**
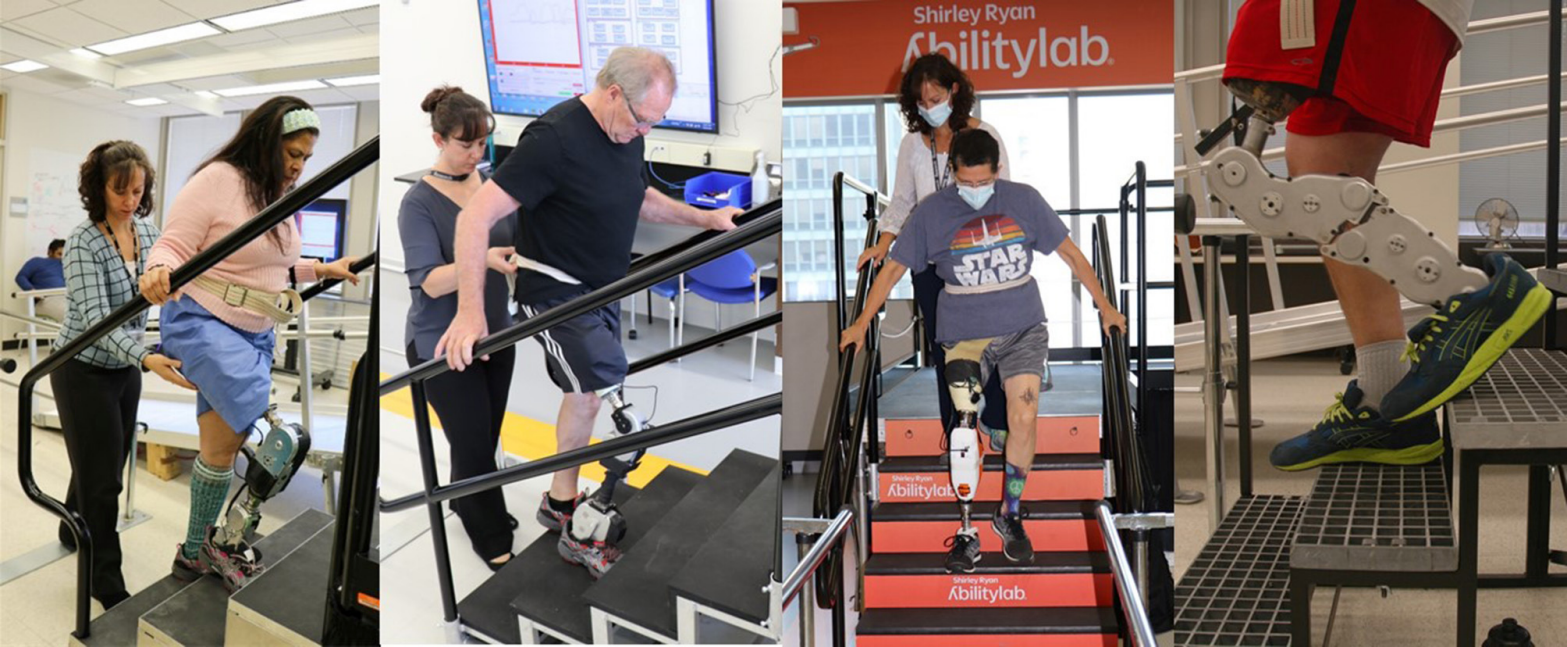
Demonstration of stair climbing with powered knee and ankle prosthesis.

Clinical goals include the ability to ascend and descend stairs (Figure 2) with unilateral upper extremity support, achieve reciprocal stepping without cueing, demonstrate consistent foot placement to achieve appropriate power initiation, and demonstrate controlled lowering during reciprocal stair descent.

##### Prosthesis Control

During stair ascent (26, 39), as the weight of the individual shift off the powered prosthesis, the device transitions to the swing phase, where the knee flexes (~90 degrees) to provide proper stair clearance. The knee, then, extends slightly and the ankle dorsiflexes (5–10 degrees) to prepare the foot for placement onto the next step. As the user shifts his/her weight onto the prosthesis, the device transitions to the stance phase, where the knee provides powered extension and the ankle provides stability as it moves under load toward the neutral position. Once the knee is fully extended, the user can position their sound limb on the next step. As they unload the prosthesis, it can provide powered plantarflexion, followed by powered knee flexion to provide clearance, and prepare for the next step.

During stair descent, as the user shifts their weight onto the prosthetic foot, the powered knee and ankle provide resistance to support the user as they “ride” the knee down for controlled descent into knee flexion and ankle dorsiflexion. As the user continues to progress through a stance of stair descent and begins to shift their load onto their sound limb, the powered prosthesis will activate the swing phase of stair descent. This will allow knee flexion and ankle dorsiflexion to clear the step and reposition for the next step.

##### Training

Reciprocal stair climbing should begin on a staircase with four or fewer stairs and bilateral handrails. Instruction begins with verbally describing the motions of the powered prosthesis during reciprocal stair ascent since most users of mechanically passive devices utilize a step to pattern of stair climbing using their sound limb to raise them to each step. Since the powered ankle can provide active dorsiflexion, users can place their whole foot onto the step and achieve a flat foot position during both stair ascent and descent, which can allow for a greater sense of stability during reciprocal stair climbing.

Training begins by ascending a single step to prepare the user for the movement and feeling of powered knee extension. While standing in front of the stair, the user shifts their weight off the prosthesis to allow the powered knee to transition into swing flexion. The user is, then, instructed to perform active hip flexion to raise the prosthesis and place the prosthetic foot fully onto the step. Physical cueing with hand placement at the lateral hip to guide the prosthetic side to assist with hip flexion and resist circumduction and vaulting. The user is instructed to push down into the prosthesis, producing pressure toward the distal/posterior portion of the socket and creating a hip extension moment. This transfer of weight onto the prosthesis is detected by the load sensor and activates the prosthetic knee extension power. Verbal instructions are given to encourage a slight forward trunk lean to assist with balance. Once the full-powered knee extension is achieved, the user will place their sound limb next to the prosthesis on the first step. Several trials of ascending one step are performed until the user feels comfortable with the movement. Stance phase stiffness parameters swing phase knee and ankle clearance, and foot position can be adjusted based on user and clinician preferences (Table 3).

**Table 3:** Stair climbing deviations, user instructions, and prosthesis parameter adjustments.

**Mode**	**Deviation**	**User instruction**	**Prosthesis setting**
Stair ascent	Poor foot placement	Cue for proper body and foot position to prepare for stair ascent	Adjust swing extension phase ankle dorsiflexion angle to achieve a foot flat position
	Decreased foot clearance	Cue to stand tall and utilize hip flex during swing phase	Increase swing phase knee flexion angle for more clearance
	Vaulting, hip hiking, circumduction	Verbal and tactile cues to improve awareness of foot position and to limit excessive hip motions.	N/A
	Inadequate support and power during stance	Cue to increase stance time on prosthesis and decrease upper extremity support	Increase stance phase knee stiffness for improved support and power into knee extension
	Insufficient swing speed	N/A	Decrease or increase rate of swing flexion
Stair descent	Unable to initiate knee flexion at initial contact	Cue for body and foot position to prepare for stair descent	Decrease stance phase knee damping to allow for easier knee flexion at initial contact
	Inadequate support during stance	Cue to increase stance time on prosthesis	Increase stance phase knee damping for increased support into knee extension during early stance phase Increase stance phase knee stiffness for increased support into knee extension during mid to late stance phase
	Poor foot placement	Cue for proper body and foot position to prepare for stair descent	N/A
	Decreased foot clearance during swing	Verbal and tactile cues to increase stance time on prosthesis and decrease UE support	Increase swing phase ankle damping

Once the user can ascend one step comfortably, he or she can progress to climbing up several steps in a reciprocal pattern, starting with their sound limb. They are reminded that shifting weight off the prosthesis will cause the knee to swing and prepare for prosthetic foot placement onto the next step. While standing behind the user, a contact guard assist with the use of the gait belt or physical palpation at the user's hips is provided and should continue to be provided to assist the users with weight-shifting and loading of the prosthesis, body position, upper extremity support, and proper foot placement. The clinician should continue to monitor swing phase clearance, quality of knee extension, foot placement, and adjust prosthesis parameters as appropriate. The amount of desired knee extension power may change throughout training as the user begins to increase their weight-bearing through the device and decrease their reliance on upper extremity support; stance phase knee stiffness can be increased to provide more support. One goal is to have users progress to only using the handrails for balance assistance (preferably only one handrail), and cues can be given to prevent the user from lifting or pulling up the step. This may take several trials to determine the appropriate power level and for the user to gain confidence in the ability of the prosthesis. If a circumduction or a sound side vaulting occurs, swing phase parameters can be adjusted to confirm stair clearance. The user's socket comfort should be monitored and modified as needed due to increased hip flexion of the amputated limb during stair ascent.

During stair descent, the method is similar to riding the knee down with a passive prosthesis, but users are reminded that for the powered prosthesis, the whole foot can remain on the step for added stability. Instructions are given to start with the prosthesis side first during descent and load the prosthesis to ride the knee down. Controlled knee flexion is achieved with stance phase stiffness and damping parameters (Table 3). When their sound side reaches the next step and they shift their weight off the prosthesis, it will swing toward knee extension in preparation for the following step allowing them to continue in a reciprocal pattern. Physical support is provided while standing behind the user with assistance at the gait belt for weight shifting onto the prosthesis and palpation at the user's shoulder to promote upright posture. Verbal instruction will be provided for foot placement on the step, and hand placement on the railing for balance stability. Clinical observations of foot placement, controlled lowering, and swing clearance should be made and prosthesis parameters can be adjusted as needed. Feedback from the user is also needed to confirm comfort and ease of stair descent.

#### Incline Walking

##### Goals

**Figure 3:**
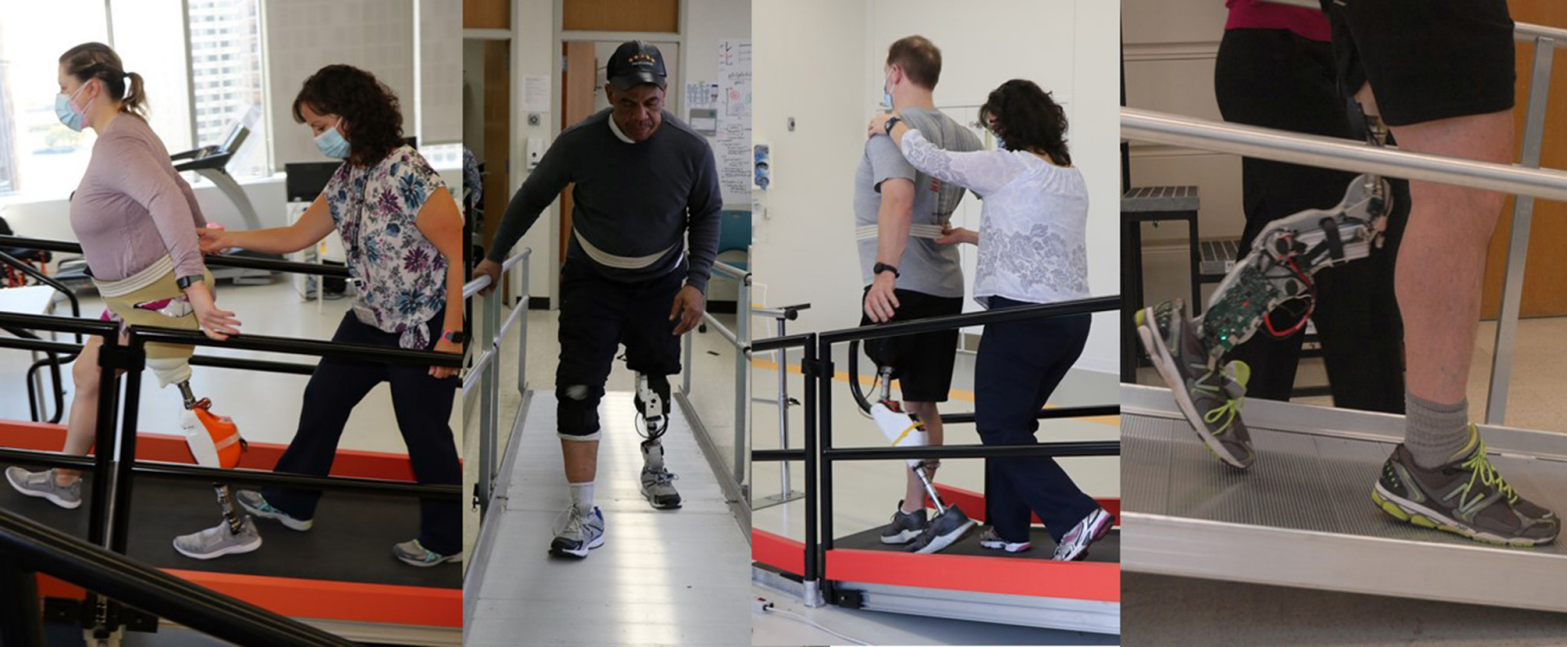
Incline walking with powered knee and ankle prosthesis.

Clinical goals include the ability to ascend and descend inclines of up to 10 degrees (Figure 3) with near equal step length, arm swing, and trunk rotation while using unilateral or no upper extremity support. Additionally, users should be able to demonstrate ramp descent with controlled lowering.

##### Prothesis Control

Control for ramp ascent mode (26, 28) is similar to level-ground walking mode. As the user loads the prosthesis through mid-stance the ankle will dorsiflex. When the ankle dorsiflexes past a pre-set angle (usually 8–10 degrees), the prosthesis will transition to the late stance phase and provide powered plantarflexion to assist with forwarding propulsion up the incline. Once the user shifts weight onto their sound limb, the powered knee flexion followed by powered knee extension will occur. Any parameter changes for level-ground walking should be transferred and used as the starting point for initial ramp ascent training.

During ramp descent, as the user loads the prosthesis, the powered knee and ankle provide resistance to support the user as they “ride” the knee down for controlled descent into knee flexion and ankle dorsiflexion. As the user progresses through stance and the ankle dorsiflexes past a pre-set angle, the prosthesis will progress through terminal stance. The swing will occur when decreasing prosthesis load is detected, as the user transfers their weight to their sound side.

##### Training

Training of incline walking begins on a slope with bilateral handrails. The user is instructed to ambulate up the ramp with bilateral upper extremity support, even step length, and a slightly forward posture to assist with propulsion up the incline. Many of the deviations seen and resolved during level-ground walking can be addressed in similar ways during incline walking (Table 4). Verbal reminders of how powered plantarflexion and powered swing extension can assist users up the ramp are beneficial, since the technology is different from their prescribed prosthesis. These motions are more pronounced during ramp ascent than in level-ground walking. During incline walking, users will likely have a greater awareness of the ankle's available range of motion into dorsiflexion, which allows the foot to remain flat on the incline during early to mid-stance.

**Table 4:** Ramp mode deviations, user instruction, and prosthesis parameter adjustments.

**Mode**	**Deviation**	**User instruction**	**Prosthesis setting**
Ramp ascent	Decreased foot clearance	Cue to stand tall and utilize hip flex during swing phase	Increase knee flexion angle during swing phase for more clearance
	Vaulting Hip hiking Circumduction	Cue and/or use a mirror to improve awareness of foot position and to decrease excessive hip motions	N/A
Ramp descent	Unable to initiate knee flexion at initial contact	Cue to increase stance time on prosthesis and decrease upper extremity support	Decrease stance phase knee damping to allow for easier knee flexion at initial contact
	Inadequate support during stance	N/A	Increase stance phase knee damping for improved support into knee extension during early stance phase Increase stance phase knee stiffness for improved support into knee extension during mid to late stance phase

During ramp descent, the user is instructed to take shorter steps during initial training to assist with weight-bearing onto the device and to “ride” the knee into flexion. If individuals are not currently using their prescribed device's stance resistance for ramp descent, this training may require several trials for them to feel comfortable putting weight through the device as the knee bends and trusting the resistance during stance. User feedback and clinician expertise are used to select parameters (adjusting knee stiffness and damping) to remove the feeling of the user “falling” down the ramp and diminish the impact on the sound limb. The clinician will observe upper extremity support and provide additional cues as the user becomes more comfortable with the powered knee stability and increase weight bearing through the prosthesis. Additional physical cues at the shoulder and hip to guide the user onto the prosthesis and direct their load down through the device to verify needed assistance for the user to adequately descend the ramp at their desired speed and support. The individual should ambulate up and down the ramp as needed while receiving cues from the clinician and parameter adjustments to achieve the clinical goals stated above.

#### Sit to and From Standing

##### Goals

**Figure 4:**
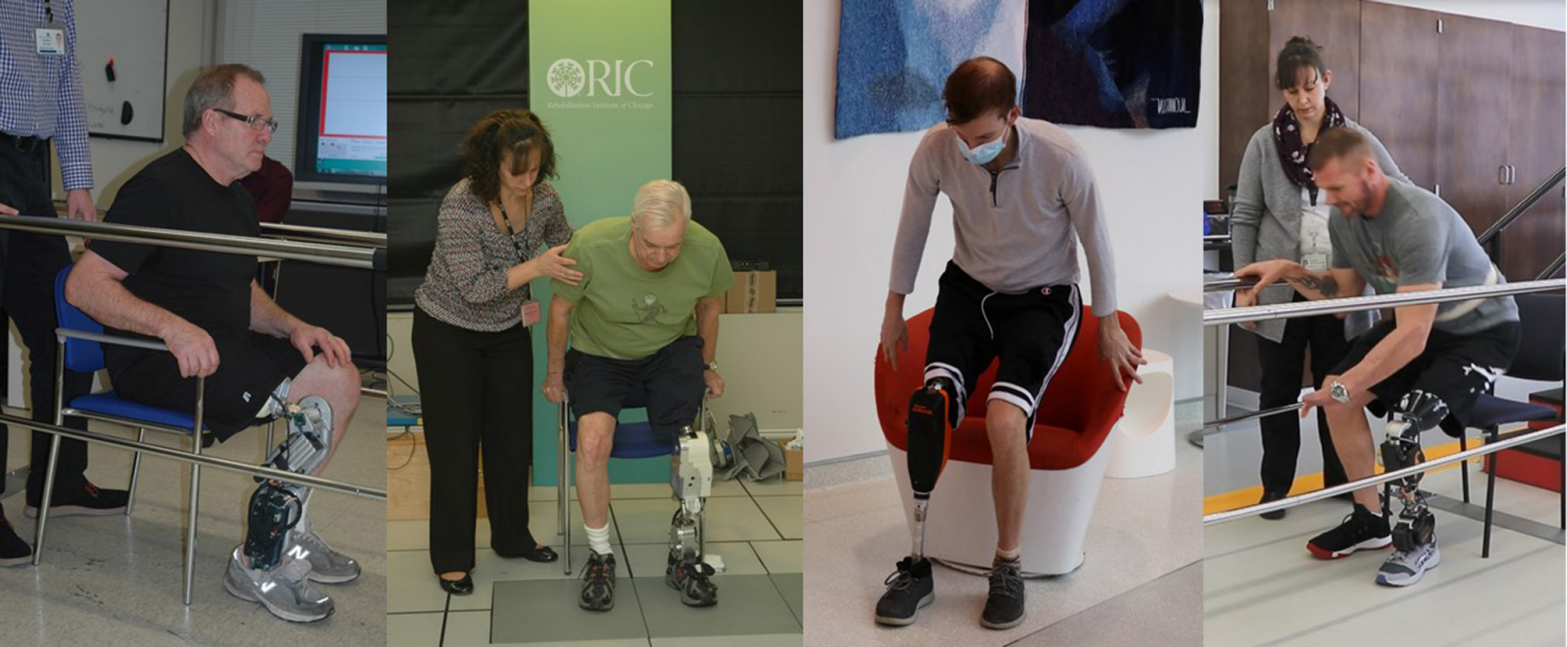
Sit-to-stand and stand-to-sit weight transfers with the powered knee and ankle prosthesis.

Clinical goals include the ability to rise from a seated position (Figure 4), with or without upper extremity support, demonstrate consistent foot placement and trunk position to achieve appropriate power initiation and comfortable standing without cueing, and demonstrate controlled lowering when completing standing to seated movements.

##### Prothesis Control

Sitting transfers are divided into four phases: stand-to-sit, relaxed sitting, sit-to-stand, and standing (29, 40). The stand-to-sit phase occurs when the user loads the prosthesis and creates a sustained knee flexion moment above a pre-set threshold. Damping and stiffness parameters in this phase will allow the user to have controlled resistance into knee flexion and ankle dorsiflexion to lower themselves to a chair. Once seated, as defined by the knee and ankle joints crossing, a pre-set flexed threshold and joint velocities are close to zero, the prosthesis transitions to the relaxed sitting phase. In the relaxed sitting phase, the knee and ankle joint remain compliant and can be easily repositioned manually by the user to a comfortable position. To initiate the sit-to-stand phase, the user shifts his/her weight onto the powered prosthesis. As load increases over a pre-set threshold, the device provides powered knee extension and powered ankle plantarflexion (from a dorsiflexed position to a neutral angle) to aid the user in rising to a standing position. Once the user is standing upright with full knee extension, the prosthesis transitions to the standing phase.

##### Training

Begin sit-to-stand training with the user standing within parallel bars with a chair of standard height, with armrests positioned closely behind them. The powered knee and ankle prosthesis offer a controlled descent by providing support throughout the full knee flexion and ankle dorsiflexion range of motion to a seated position. While standing, they are instructed to have equal weight through each leg and apply a knee flexion moment by attempting to sit with active hip flexion and a forward trunk lean. It is beneficial to provide verbal instruction on trunk position and physical assistance through the user's hips and spine to encourage equal weight bearing through the lower extremities while the prosthesis flexes to a seated position. The user may utilize the armrests for balance if needed while transferring to the chair. The clinician should monitor foot and trunk position, loading through the device, and rate of controlled flexion and adjust parameters as needed (Table 5). For sitting down, the knee and ankle joint damping parameters can be increased or decreased for more or less support, respectively. As the user becomes comfortable with the movement into sitting, they may start to increase their load onto the prosthesis and parameters can be further adjusted for increased stiffness.

**Table 5:** Sit-to-stand and stand-to-sit deviations, user instructions, and prosthesis parameter adjustments.

**Mode**	**Deviation**	**User instruction**	**Prosthesis setting**
Stand to sit	Unable to initiate sitting	Cue for active hip flexion and equal weight bearing between limbs for increased load onto the prosthesis	Decrease axial load threshold
	Inadequate support during sitting	N/A	Increase or decrease knee damping to provide more or less support, respectively
Sit to stand	Unable to initiate standing	Cue to increase forward trunk position and load onto the prosthesis	Decrease axial load threshold to initiate knee extension power
	Inadequate support or power during standing	Cue for equal weight bearing between limbs for increased load onto the prosthesis	Increase stance phase knee stiffness for improved support and power into knee extension

Once the user is seated and in the relaxed sitting phase, they may adjust the prosthesis passively to their desired position for sitting or to prepare to stand. The available range of motion at the knee and ankle joint allows the user to scoot toward the edge of the chair, align their feet evenly for the equal load on both limbs and maintain a flat foot position. This position will enable bilateral limb muscle activation and improved pelvic symmetry for a smoother, more efficient transition to standing. For users who have difficulty initiating load in the prosthesis to facilitate powered knee extension, enabling visual feedback of the amount of load in their prosthesis allows both users and clinicians to become accustomed to the amount of forward lean and load that is needed to initiate stand without engaging power at the device may be helpful.

Clinicians should evaluate the ease of initiating standing along with the rate and movement quality of rising to stand, while prosthesis parameters can be adjusted as needed (Table 5). Tactile cues are provided to the user along their torso to encourage forward lean, and with the gait belt to pull the user toward their prosthetic side to increase weight-bearing through the prosthesis while standing up. Verbal cues and demonstration of proper foot positioning and posture will assist for successful sit-to-stand transitions. If the user is having difficulty initiating sit to stand, and cues don't resolve the issue the axial load threshold can be decreased for an easier transition. Stance phase knee stiffness can be increased for increased support and power into knee extension to achieve standing. If there continues to be user hesitation to load the prosthetic foot from sitting to standing, training techniques may include staggering their feet; placing the prosthetic foot slightly behind the sound foot will force an increased weight bearing on the prosthesis. An alternative method is to provide support in front of the user and guide their upper extremities and trunk forward and slightly toward their prosthetic side. By being present in front of the user, they feel more secure and may allow themselves to lean forward over their toes to increase load through the device.

## Results

All 30 participants were successful in powered leg-fitting and ambulation over level-ground (see Supplementary Video 1). Twenty-seven participants were able to continue with training sessions and became successful in independent ambulation of all other functional modes after participating in 1–3 sessions of 1–2 h in length. Three of the participants did not continue with additional powered leg ambulation training. Two subjects (TF02 and TF15) were unable to continue with the training of stair climbing and ramp ascent due to fatigue and tolerance of the weight of the powered prosthesis required for stair ascent and incline walking. Another subject (TF17) was unable to continue due to the cognitive task of reciprocal stair climbing; he required multiple cues for foot placement and a residual limb control required for stair climbing.

**Figure 5:**
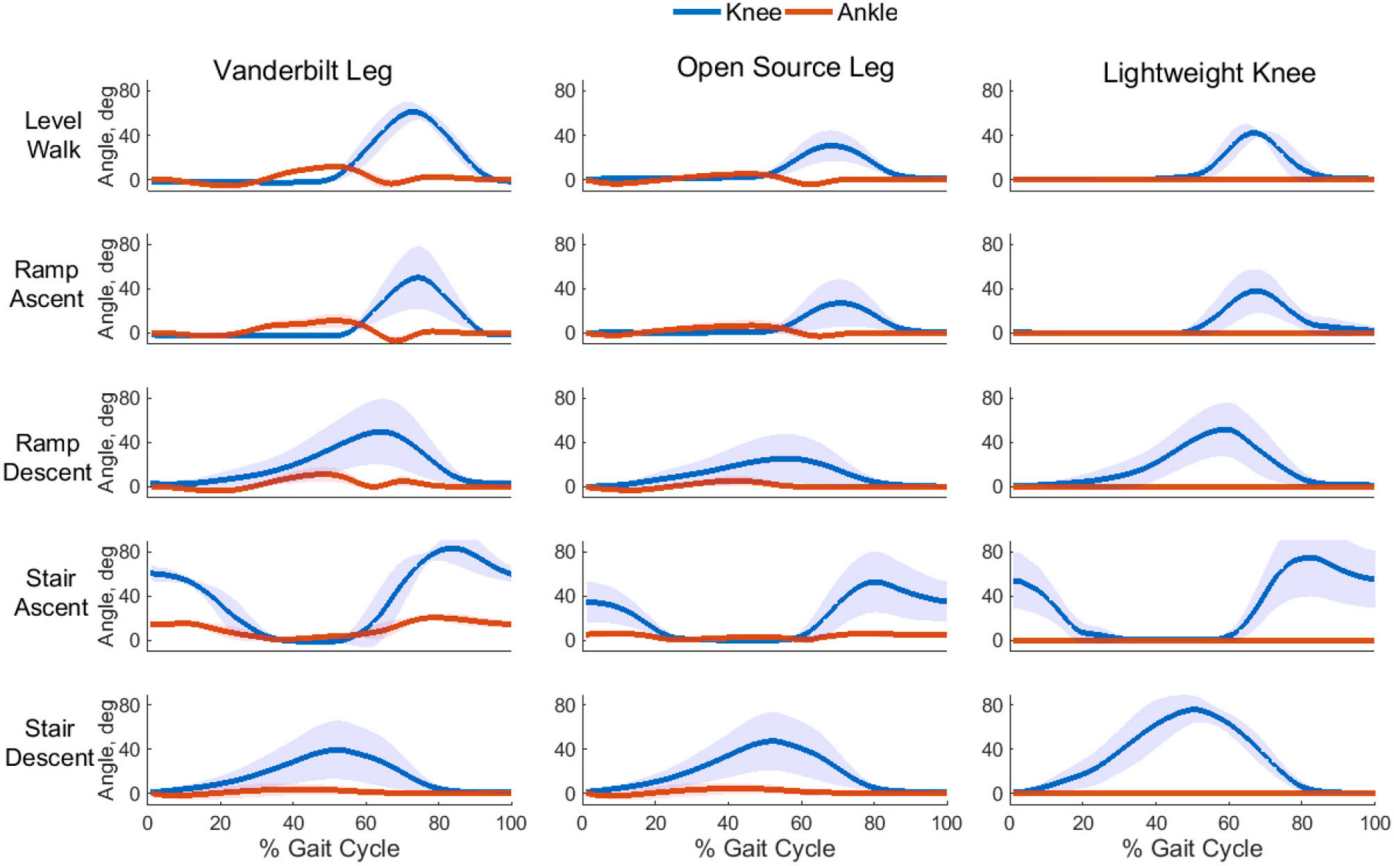
Averaged powered prosthesis knee and ankle angles during all ambulation modes for all participants. Shaded regions represent +/– one standard deviation (SD).

Several of the subjects were trained on multiple powered legs throughout the development: 27 of the participants were trained on the Vanderbilt powered knee-ankle prosthesis, 14 of the participants were trained on the OSL, and 11 of the participants were trained on the lightweight robotic knee with a passive ankle [low-profile Vari-Flex foot (41)]. Nine of the participants had the opportunity to train on all 3 powered leg prostheses. Figure 5 outlines averaged prosthetic leg knee and ankle kinematics across all trained ambulation modes.

## Discussion

The current market for transfemoral amputees and the passive prosthesis is very focused on the functional level of the amputee to which device they would be best paired with for success. The functional level is decided by the clinical team based on the amputee's current and potential functional status. Many of the participants enrolled in our study are unable to climb a staircase reciprocally or descend an incline forward with the use of their currently prescribed prosthesis but were successful using a powered prosthesis. Powered knee and ankle prostheses have the potentially to truly improve the amputee's activities of daily living based on proper training and device control and development.

Over the past 10 years of developing these training techniques, participant feedbacks included that the powered prosthesis felt very different from their currently prescribed prosthesis, was more intuitive to use, allowed them to “walk without thinking at each step,” and did not have to actively or forcefully move their hip forward to advance the prosthesis. Once trained, users were able to ambulate very comfortably as evident by holding conversations, carrying items, and navigating in tight spaces without noticeably increased effort. Following the development of this training, the majority of users, who are now being trained, are successful in ambulating across all five activity modes within 1–3 sessions of 1–2 h each. Occasionally a few participants needed 1–2 additional sessions to address socket fit or socket suspension issues due to the weight of the powered leg being greater than their prescribed prosthesis, and/or becoming more comfortable with the power and movements of the device.

Ambulation training was based on allowing the user to walk as they did before their amputation. Learning to climb stairs with a reciprocal pattern and/or stand up from a chair incorporating their residual limb and prosthesis was at first both a physical change (e.g., the prosthetic side would lead on every other step during stair climbing) and a cognitive change (e.g., users had to remember to engage their prosthetic side while standing up from a chair). While only higher level and very active users can demonstrate reciprocal stair climbing with a passive knee unit, since it requires extensive residual limb strength and stability, all participants in this study who tried the powered prosthesis were successful climbing with a reciprocal gait. A few participants required additional cueing for stair climbing for appropriate loading of the prosthesis and trunk position. Often this was during initial stair ascent training and after 2–3 successful steps, they begin to have more trust in the movements, increase their load through their socket, lessen their upper extremity support, and relax their trunk position into a more natural posture. Participants often expressed excitement to have the ability to ascend several stairs and even staircases with ease due to the active power provided by the prosthesis. The users responded positively when given the ability to rely on the prosthesis when rising from a chair and reported decreasing load on their sound limb and support on the arms of the chair. Positive reactions were also expressed when walking up a large incline since the effect of the ankle power when ascending a ramp was felt immediately. Several individuals stated they were able to walk up the incline faster, with improved ease, and with less (or no) reliance on upper extremity support. Anecdotally, it was observed for some users that sound side vaulting was minimized or diminished during level-ground and incline walking with the powered knee and ankle prosthesis without any specific instructions or cueing when walking.

Only a small percentage of participants (3 of 30) were unable to complete full training of all ambulation modes with a powered leg prosthesis. Two individuals were independent with level-ground ambulation, but experienced difficulty when needing to lift the prosthesis either upward for stair ascent or forward up an incline. The passive lifting required by the user's hip and abdominal muscles was difficult for these two participants who had short residual limbs, resulting in an increased load onto their limb and hip musculature. Additionally, one individual was unable to perform the cognitive task of reciprocal stair climbing. Although he may have been able to eventually re-learn this task, additional sessions in the research environment were not available at the time. While the majority of subjects provided positive feedback while using the powered knee and ankle prosthesis, several reported the desire for the leg to be lighter, quieter, and have water-resistant capabilities to allow them to use the device with all desired activities.

The kinematic data shown in Figure 5 were based on user and device testing across several years of research on three different powered leg prostheses, multiple users on each device, and at different times during powered leg training development. These data in this paper intend to demonstrate kinematics of successful use across multiple modes of ambulation and not necessarily to compare between devices. Most of the differences, if not all, identified in Figure 5 can be explained by the differences in hardware and/or improvements in control that developed over time. Additionally, the testing of these devices was rather sequential: testing of the Vanderbilt Leg spanned from 2011 to 2018, Open Source Leg from 2017 to 2021, and the Lightweight Knee from 2019 to 2021. For example, the Vanderbilt Leg prosthesis had 70 degrees range of motion available (15) at the ankle, whereas the Open Source Leg only had 30 degrees range of motion available (42). Therefore, ambulation on the Vanderbilt Leg, compared to the Open Source Leg, could take advantage of this increased range of motion including increased stance phase dorsiflexion during stair and ramp descent and late-stance powered plantarflexion during level-ground and incline walking. Additionally, as we became more proficient in our control settings for powered leg prostheses, we realized that for adequate toe clearance, we did not need to flex the knee as much during the swing phase (e.g., in Figure 5, comparing maximum knee flexion during the swing phase of walking with the Vanderbilt Leg to that of both the Open Source Leg and Lightweight Knee). Had we identified this improvement earlier in our development, we could have easily adjusted with the Vanderbilt Leg to result in similar knee kinematics between the legs and hence, similar swing clearance for the users.

These data did, however, help in developing this training protocol of how to teach individuals with a transfemoral amputation and how to walk on a powered leg prosthesis. Simultaneously, as we were learning to control a powered prosthesis, we were developing the appropriate clinical training cues based on the feedback received. The speed at which our users became accustomed to the device was quicker than initially expected (e.g., we progressed training to inclines and stairs much sooner than anticipated).

We now have a training protocol for powered lower limb prostheses that we hope will assist other research groups, including our own, in providing training for these devices before performing studies that involve functional performance outcome measures and/or biomechanics. These comparative studies will be important to assist in identifying when (e.g., which ambulation tasks) and where (e.g., ankle only, knee only, or both knee and ankle) users can best take advantage of the power available from these devices. Although the data included in this study cannot make these comparisons, we were surprisingly successful in training users from the various demographic backgrounds; participants with a wide range of time since amputation (9 months−47 years), height (160–193 cm), weight (52–111 kg) all had similar training time. A female who was 160 cm and 52 kg and 7 years post-amputation completed all modes as easily as a male who was 186 cm, 111 kg, and 39 years post-amputation. Both users were able to ascend stairs and ramps with the same instruction and ease with initial parameter and joint power settings based on their weight.

Ambulation and negotiation of the various activity modes were also successful across individuals with a variety of suspension systems, provided that the setup used could accommodate the increased weight of the powered device. Participants that used a pin-locking liner did display increased rotation during the swing, likely due to the active knee power. Since we did not change individuals' primary method of suspension, the addition of a Total Elastic Suspension belt for these users eliminated the rotation. A TES belt for secondary suspension was also necessary for users who presented with shorter residual limbs. Users with shorter residual limbs often required supplementary training to properly lift and load the powered prosthesis. Palpation at the user's hips and lower back to provide tactile cues to incorporate hip flexors and abdominal muscles, decrease posterior lean aided for proper prosthetic side foot placement on a stair to properly load their socket using active hip extension when ascending stairs, and complete sit to stand transitions allowed for the prosthesis to respond with active knee extension.

While these training methods were developed using three different powered leg prostheses [i.e., Vanderbilt Powered Knee and Ankle Prosthesis (15), the Open Source Robotic Leg (37), and the lightweight hybrid robotic knee (38)] in a rehabilitation facility environment, we expect most of the methods to transfer to similar powered lower limb devices. Additional training techniques may be necessary for outdoor/uneven terrain ambulation and obstacle avoidance for participants to function independently in their home environment. These methods were developed while training high-level (K3 and K4) ambulators with non-vascular reasons for amputation. The duration or frequency of training may change for K2 ambulators. Additional cues may be needed to load the prosthesis during ascent activities secondary to decreased strength or balance deficits or to incorporate an assistive device. Finally, since training occurred on a prosthesis that is not yet clinically/commercially available, all participants attended training sessions and, then, returned to their prescribed and passive prosthesis at the end of the research sessions.

## Conclusions

As powered lower limb devices become more clinically available, they will continue to challenge physical therapy practice in terms of instructional gait and advanced mobility training. Through this training protocol, clinicians can gain a better understanding of the technical aspects of how the device is controlled, as well as the benefits and limitations, to provide better training and outcomes for users of lower limb prostheses across multiple modes of ambulation. Physical therapists should be encouraged to study and understand these devices through education from prosthetists, manufacturers, and published research studies and protocols.

## Data Availability Statement

The raw data supporting the conclusions of this article will be made available by the authors, without undue reservation.

## Ethics Statement

The studies involving human participants were reviewed and approved by Northwestern University Institutional Review Board. The patients/participants provided their written informed consent to participate in this study. Written informed consent was obtained from the individual(s) for the publication of any potentially identifiable images or data included in this article.

## Author Contributions

LH conceived of the original idea and supervised the project. SF and AS performed the subject training, data collection sessions, and developed the subject training process. AS and LH analyzed the data. SF, AS, and LH wrote and edited the manuscript. All authors contributed to the article and approved the submitted version.

## Funding

Funding for this report was provided by the US Army's Telemedicine and Advanced Technology Research Center (TATRC) contract WW81XWH-09-2-0020, the US Army's Joint Warfighter Program contract W81XWH-14-C-0105, the National Institute of Health (NIH) R01 HD079428-02 and the National Institute on Disability, Independent Living, and Rehabilitation Research (NIDILRR grant number 90REGE0003. NIDILRR is a Center within the Administration for Community Living (ACL), Department of Health and Human Services (HHS). The contents of this article do not necessarily represent the policy of NIDILRR, ACL, or HHS, and you should not assume endorsement by the Federal Government.

## Conflict of Interest

The authors declare that the research was conducted in the absence of any commercial or financial relationships that could be construed as a potential conflict of interest.

## Publisher's Note

All claims expressed in this article are solely those of the authors and do not necessarily represent those of their affiliated organizations, or those of the publisher, the editors and the reviewers. Any product that may be evaluated in this article, or claim that may be made by its manufacturer, is not guaranteed or endorsed by the publisher.

## Supplementary Material

Supplementary Video 1 Clinical training goals and example videos of individuals learning to use a powered prosthesis for level and incline walking, stair climbing, and during weight transfers.
