# Corrigendum: Functional mobility training with a powered knee and ankle prosthesis

**DOI:** 10.3389/fresc.2022.1004110

**Published:** 2022-08-09

**Authors:** Suzanne B. Finucane, Levi J. Hargrove, Ann M. Simon

**Affiliations:** ^1^ Shirley Ryan AbilityLab, Chicago, IL, United States; ^2^ Department of Physical Medicine and Rehabilitation, Feinberg School of Medicine, Northwestern University, Chicago, IL, United States; ^3^ Department of Biomedical Engineering, McCormick School of Engineering and Applied Science, Northwestern University, Chicago, IL, United States

**Keywords:** physical therapy, above-knee amputation, ambulation, robotic prosthesis, rehabilitation, artificial leg, prosthesis training, transfemoral amputation

A corrigendum on Functional mobility training with a powered knee and ankle prosthesis by Finucane SB, Hargrove LJ and Simon AM (2022). Front. Rehabilit. Sci. 3:790538. doi: 10.3389/fresc.2022.790538

**Incorrect Funding**

In the published article, there was an error in the Funding statement. The funding statement was missing an additional funding source as listed below. Original text: Funding for this report was provided by the US Army's Telemedicine and Advanced Technology Research Center (TATRC) contract WW81XWH-09-2-0020, the US Army's Joint Warfighter Program contract W81XWH-14-C-0105, and the National Institute of Health NIH R01 HD079428-02.

The correct Funding statement appears below.

**Funding**

Funding for this report was provided by the US Army's Telemedicine and Advanced Technology Research Center (TATRC) contract WW81XWH-09-2-0020, the US Army's Joint Warfighter Program contract W81XWH-14-C-0105, the National Institute of Health (NIH) R01 HD079428-02 and the National Institute on Disability, Independent Living, and Rehabilitation Research (NIDILRR grant number 90REGE0003. NIDILRR is a Center within the Administration for Community Living (ACL), Department of Health and Human Services (HHS). The contents of this article do not necessarily represent the policy of NIDILRR, ACL, or HHS, and you should not assume endorsement by the Federal Government.

The authors apologize for this error and state that this does not change the scientific conclusions of the article in any way. The original article has been updated.

